# Treatment Seeking Behavior, Treatment Cost and Quality of Life of Head and Neck Cancer Patients: A Cross-Sectional Analytical Study from South India 

**DOI:** 10.31557/APJCP.2021.22.9.3023

**Published:** 2021-09

**Authors:** Arvind Kumar, Swaroop Kumar Sahu, Gunaseelan Karunanithi, Karthik Balajee Laksham

**Affiliations:** 1 *Deputy General Manager, Cankids Kidscan, Delhi, India. *; 2 *Department of Preventive and Social Medicine, Jawaharlal Institute of Postgraduate Medical Education and Research, Puducherry, India. *; 3 *Department od Radiotherapy, Jawaharlal Institute of Postgraduate Medical Education and Research, Puducherry, India. *; 4 *Department of Community Medicine, Jawaharlal Institute of Postgraduate Medical Education andamp; Research (JIPMER), Karaikal, Puducherry, India. *

**Keywords:** Head and neck cancer, FACT, HandN, quality of life, treatment cost, treatment seeking behavior

## Abstract

**Background:**

Head and neck cancer constitute one-third of all cancers. Due to the complex nature of Head and Neck Cancer treatment, expenditure on cancer treatment are higher and the Quality of Life of patients is also compromised. The objectives of the study were to determine the time taken by patients for seeking care from registered medical practitioners, time to definitive diagnosis and treatment initiation, expenditure incurred, and Quality of Life. Methods: The present study was a cross-sectional descriptive involving outpatient with head and neck cancer reported to the department of radiotherapy, regional cancer center, JIPMER. The quality of life was assessed using validated FACT-H and N scales.

**Results:**

The preferred first contact for seeking care for most was the private sector (52%). The median (IQR) presentation interval, diagnostic interval, and treatment initiation interval were 36.5 (16 - 65.7), 14 (7 - 31.5), and 65.5 (45 - 104) days respectively. The average indirect cost incurred was INR 8424 (4095-16570) in JIPMER, which was spent over an average duration of 240 days. The median (IQR) wage loss by the patients and/or caregivers was INR18000 (5250-61575). The source of expenditure was mainly from their family savings (56%). Functional well-being was severely impaired. The patients with occupation, head of the family, and early stage of cancer had a statistically significant quality of life.

**Conclusion:**

The majority of the patients were diagnosed in the regional cancer center, JIPMER although their preferred first point of contact was private practitioners. The average time interval from diagnosis to treatment initiation was more than two months. The expenditure during the treatment was mainly because of indirect cost and wage loss. The functional quality of life was severely impaired for the majority of the cases.

## Introduction

Cancer is the second most common cause of death following cardiovascular disorders (Roth et al., 2018). Globally 14.1 million new cancer cases and 8.2 million deaths occur every year, and 32.5 million people are living with cancer (World Health Organization, 2014b). Head and Neck cancers (HNC) account 23 % of all cancer cases (Dikshit et al., 2012) They arise from the mucosal lining (squamous cell), and include Oral and Oropharyngeal carcinoma, Nasal and Nasopharyngeal carcinoma and Hypopharyngeal carcinoma (Shah and Lydiatt, 1995). HNC is the highest occurring cancer among the males and third highest in females. The Indian Council of Medical Research (ICMR), has estimated that approximately 0.20 to 0.25 million new Head and Neck cancer patients are diagnosed every year (National Cancer Registry and Programme Indian Council of Medical Research, 2016) and this constitutes about 30% of all incident cancers. India has the highest rate of oropharyngeal cancers accounting for 30-40% of all cancers (National Cancer Registry and Programme Indian Council of Medical Research, 2016) and its mortality was 18% in males and 7% in female (World Health Organization 2014a). The International Agency for Research on Cancer (IARC) estimated that the incidence of cancer will sharply increase by 50% in 2020 (Ferlay and Soerjomataram, 2015) and the reasons behind this are increasing life expectancy and aging population worldwide. This prediction was made considering the current trend of increasing tobacco consumption and the adoption of an unhealthy lifestyle (World Health Organization, 2014b). 

Early diagnosis and timely initiation of treatment of Head and Neck cancers improves survival, lower the cost of care and results in retention of a better quality of life.(Kumar et al., 2012) Most patients experience a drop in their income while undergoing diagnosis and treatment. During treatment indirect cost is a major burden to the patients, increasing their financial stress and can drive many families to economic catastrophes (Kavosi et al. 2014; Sharp and Timmons, 2010; Nair et al., 2013). In India, Public health facilities provide free or subsidized treatment. Patients usually initiate care in the private sector because of perceived better treatment and perceived better chances of survival before they start seeking care in a public facility (Nair et al., 2013). 

Quality of life (QOL) is a multidimensional concept measuring the physical, social/ familial, emotional, and functional wellbeing of an individual (Webster et al., 2003). HNC can affect the quality of life of an individual by affecting the normal speech, breathing, and eating and disfigurement (Bernier et al., 2004). In India, the literature on the QOL of patients with HNC and time took for seeking care, getting diagnosed, and treated is limited.

Against this background, we planned to conduct a study among the head and neck cancer patients who attended department of radiation oncology; the objectives were 1). to determine the time intervals in presentation, diagnostic, and treatment initiation and various pathways of the care sought before reaching our tertiary care facility, 2). To estimate their treatment cost and sources of their health expenditure and 3). Socio-dempographic and clinical factors associated with quality of life (QOL).

## Materials and Methods

*Study design*

The study was a hospital-based cross-sectional analytic study conducted among patients who attended department of radiation oncology.

*Setting*

The study was carried out in the Jawaharlal Institute of Postgraduate Medical Education and Research, an institute of national importance located in Puducherry. Puducherry is one of the eight union territories of India. The Union Territory of Puducherry lies in the southern part of the Indian Peninsula. The population of Puducherry was 1.2 million as per the 2011 census. The RCC offers services to around 3000 new cancer patients every year of whom 990 suffered from head and neck cancer. The RCC now includes the Departments of Radiotherapy, Medical Oncology, and Surgical Oncology. Cancer treatment to patients in JIPMER is mostly free. The cancer patients are also referred from other eastern and southern Indian states. The patients from the nearby state of Tamil Nadu avail service through the Chief Minister’s Comprehensive Health Insurance Scheme, under which individuals belonging to annual income less than INR 72,000 (~1006 $) can avail free treatment services. In 2002, the Department of Radiotherapy was upgraded to Regional Cancer Centre (RCC). Approximately 1,200 patients availing advanced diagnostic and treatment services including radio-diagnosis, pathology, medical oncology, surgical oncology and radiotherapy. The fee for consultation and investigation is free for all patients.

*Selection of patients*

The study participants included all newly registered and follow-up adult patients with head and neck cancer seeking treatment at Radiotherapy department, JIPMER between 1^st^ August 2016 to 30^th^ September 2016. Convenient sampling was adapted for the study. A total of 195 adult patients with head and neck cancer who attended Radiotherapy OPD during the period of data collection were approached for inclusion in the study. Among them, 192 patients who gave consent were included in the study. All diagnosed head and neck cancer patients with date of definite treatment were recruited consecutively. Patients who were diagnosed for more than three years were excluded. 

*Data collection and processing*

Sociodemographic details, clinical and medical history were extracted from the patient’s case sheet. The date of diagnosis and treatment initiation were extracted from the patient’s current and previous hospital records. The hospital record files of the patients were retrieved from medical record dapertment. The files number were noted based on the eligibility criteria and eligible patients were approached for inclusion in the study. Study participants were interviewed after completeion of their consultation with treating physician or procedures, the participants were interviewed in a separate room ensuring privacy. Information on date of recognition of symptom, type and number of health care providers visited, date of visit, date of definitive diagnosis and treatment were collected using a self administered structured questionnaire. 

Three types of time interval were elicited, i.e., the first time interval was considered from onset of symptom till they sought their first consultation from a registered medical practitioner (presentation interval), the second time interval was from the time of consultation with the registered medical practitioner till definitive cancer diagnosis was made (Diagnostic interval) and the third one was from the time of diagnosis till initiation of definitive cancer treatment (Treatment initiation interval). The expenditure on consultation, investigation, and treatment was considered as a direct cost, and the expenditure on transport, food, and lodgement was considered as an indirect cost. All costs incurred by patients were elicited for the whole duration of their illness preceding the date of the interview and recorded in Indian National Rupees (INR). 1 dollor (US) is equals to INR 66.9.

*Study tool*

The instrument used for assessing Quality of Life (QOL) was the validated Tamil version of the Functional Assessment of Cancer Therapy (FACT) scale (version 4). The patient’s responses were marked on the scale of 0 to 4; as was most appropriate to their condition in the past seven days. Negatively stated items were reversed by subtracting the responses from “4”. All subscale items were summed to derive the total score. Four subscales, i.e., Physical, Social, Emotional, and Functional (27 items) together constituted FACT-General (FACT –G) summary score. Specific questions related to head and neck were added to above mentioned four subscales in the FACT-HandN scale having 39 items. The total scores were divided into groups of three based on the absolute number (Fisch et al. 2003). The low score was considered as a severe impairment, moderate score as moderate impairment, and high score as low impairment. The higher the score, the better was the QOL.

QOL was measured using the Functional Assessment of Cancer Therapy (FACT) general and specific. 

Prorated subscale score = [Sum of item score] × [N of items in subscale] ÷ [N of items answered]

*Statistical methods and Analysis*

The data was entered using EpiData Entry client (v2.0.9.25) and analyzed using EpiData Analysis version 2.2.2.183 (EpiData Association, Odense, Denmark) and SPSS version 19.

Sociodemographic, clinical, and treatment variables were expressed as frequency and proportions. Continuous variables like time intervals, direct and indirect cost were expressed as median with Interquartile Range (IQR). The refernce time point for the economic cost to the patient and QOL was data collection period. 

QOL subscale and summary scale were expressed as Mean and Standard Deviation (SD). The association between the exposures and the FACT summary scale was analyzed using Kruskal Walis ANOVA and independent t-test. Statistical significance was considered as a p-value of less than 0.05.

*Ethical approval*

The study was approved by the Institute’s Scientific Advisory Committee and Ethical clearance was obtained from the Institute Ethics Committee (Human studies), before the start of the study [project no JIP/IEC/SC/2016/29/890]. Informed written consent was obtained from all the patients. The interview was conducted in a separate room and confidentiality of the patients information was maintained throughout the study. The patient information sheet and written informed consent in the regional languages was obtained from the particpants before conducting the interviews.

**Table 1:** Distribution of Socio-demographic and Clinical Characteristics of Head and Neck Cancer Patients attended at Radiation Oncology (N=192).

Characteristics	Frequency (n)		Proportion (%)
Age			
<44 years	30		15.6
45-59 years	90		46.9
>60 years	72		37.5
Gender			
Male	128		66.7
Female	64		33.3
Religion			
Hindu	165		85.9
Christian	19		9.9
Muslim	8		4.2
Marital status			
Married	166		86.5
Widow	13		6.8
Widower	7		3.6
Single	6		3.1
Residence			
Rural	133		69.3
Urban	59		30.7
Type of family			
Nuclear	123		64.1
Joint	69		35.9
Education			
No Formal Education	74		38.5
Primary	53		27.6
Middle school	40		20.8
High school	18		9.4
Higher secondary	7		3.6
Occupation			
Unemployed	142		74
Employed	34		17.7
Home maker	16		8.3
Head of family			
Self	132		68.8
Others	60		31.3
State			
Tamil Nadu	157		81.8
Pondicherry	29		15.1
Others*	6		3.1
Socio economic status†			
I (>6323)	2		1
II (3161-6322)	14		7.3
III (1897-3160)	46		24
IV (948-1896)	77		40.1
V (<947)	53		27.6
Oraland Oropharynx	146	76		
Larynx and Hypopharynx	34	17.7		
Others‡	12	6.3		
Stages				
Stage I and II	31	16.1		
Stage III	37	19.3		
Stage IV	124	64.6		

Mean (SD) age, 54.92 (10.58); Range, 28-85; *Others include Karnataka, West Bengal, Jharkhand, Andhra Pradesh and Andaman Nicobar Island. †Socio-economic status of the patients calculated according to the Modified BG Prasad scale (CPI (IW) Base 2001=100 Monthly Index). Because study participants were mixed, so all India general Index (277) considered for calculating socioeconomic status.‡Others include nasal (n=1), Nasopharynx (n=7), Thyroid (n=3) and Parotid gland (n=1).

**Table 2:** Distribution of Different Time Intervals (in days) among Head and Neck Cancer Patients at Department of Radiation Oncology During August-September 2016

Time interval (in Days)	Median (IQR)
Presentation interval (N=192)	36.5 (16 - 65.7)
Diagnostic interval (N= 172)*	14 (7 - 31.5)
Treatment interval (N=190) †	65.5 (45 - 104)

* 20 patients got diagnosed before reporting to study centre. Their median time interval till definite diagnosis was 10.5 (IQR, 4.75-15.75)

†2 patients had initiated their treatment prior to reporting to the study centre.

**Table 3:** Total Indirect Cost Incurred by all Head and Neck Cancer Patients and Attendants ever Reported in Different Facilities in INR

Indirect Cost Med (IQR)	Reported to radiation oncology N=192	Reported to Govt/ Medical college/ ESIC* N= 65	Pvt hospital/ ENT†/ Dentist N= 100	Reported to Quack‡ N= 10	Total of All facilities
Transport	5400	200	120	0	1300
	2400-10500	55-475	32.5-300	0-145	140-5820
Food	2800	0	0	0	500
	1125-5262.	0-290	0-100		0-3100
Total Indirect cost*	8424	200	135	842.5	2300
	4095-16570	55-915	42.5-500	0-3600	150-9100
Days Med (IQR)	240	14	7	30	
	116-607	(5-21)	(1-20)	(20-142)	

*ESIC-Employees' State Insurance Corporation hospital; † Ear Nose and Throat specialist; ‡Quack- an unqualified person who claims medical knowledge or other skills.

**Table 4:** Socio-demographic and Clinical Factors Associated with Quality of Life among Head and Neck Cancer Patients at Radiation Oncology (N=192)

Characteristics	FACT-G		FACT-HandN	
	Mean (SD)	p-value	Mean (SD)	p-value
Age		0.4		0.5
<44	55.3 (12.5)		72.7 (15.3)	
45-59	52.9 (11.5)		69.8 (13.5)	
>60	51.6 (13.7)		69.2 (16.2)	
Gender		0.26		0.03
Male	67.7 (8.4)		85.8 (11.9)	
Female	66.3 (8.4)		81.9 (11.3)	
Marital status				0.57
Married	67.3 (8.4)	0.59	84.6 (11.9)	
Widow	65.2 (7.8)		80.6 (10.1)	
Widower	68.2 (8.8)		86.8 (11.8)	
Single	70.8 (8.8)		87.2 (12.5)	
Residence		0.75		0.67
Rural	67.2 (8.9)		84.3 (12.1)	
Urban	67.5 (7.1)		85.1 (11.3)	
Type of family		0.27		0.18
Nuclear	68.2 (7.6)		83.7 (12.2)	
Joint	66.8 (8.8)		86.0 (11.0)	
Education		0.2		0.08
No Formal Education	67.1 (9.0)		83.8 (12.1)	
Primary	67.3 (7.2)		84.2 (9.9)	
Middle school	66.6 (7.5)		84.3 (11.7)	
High school	66.5 (6.7)		84.3 (10.4)	
Higher secondary	74.8 (15.5)		96.9 (20.4)	
Occupation		0.02		0.01
Unemployed	66.5 (7.4)		83.5 (10.6)	
Employed	70.8 (10.8)		89.8 (13.9)	
Home maker	66.8 (9.7)		81.9 (14.4)	
Head of family		0.05		0.01
Self	68.0 (8.79)		85.8 (12.3)	
Others	65.6 (7.4)		81.5 (10.3)	
Socio economic status		0.2		0.7
I	63.3 (4.0)		82.3 (12.5)	
II	72.2 (13.3)		88.5 (17.7)	
III	66.7 (7.9)		84.2 (12.2)	
IV	66.7 (7.0)		84.4 (10.3)	
V	67.4 (9.1)		83.9 (12.1)	
Site of Cancer		0.05		0.04
Oral and Oropharynx	52.4 (12.4)		69.7 (14.9)	
Larynx and Hypopharynx	51.5 (13.1)		67.8 (13.9)	
Others*	61.2 (10.3)		79.9 (13.1)	
Stages of cancer		0		0
Stage I +II	58.1 (15.0)		76.9 (16.8)	
Stage III	57.7 (7.1)		75.7 (12.6)	
Stage IV†	40.2 (8.7)		66.6 (13.8)	

*Others include Nasal, Nasopharynx, Parotid, and Thyroid; †Stage IV include IVA, IV B and IV C; Test used for estimation of association are Independent t test and one-way ANOVA with post hoc test (Tukey).

**Figure 1:**
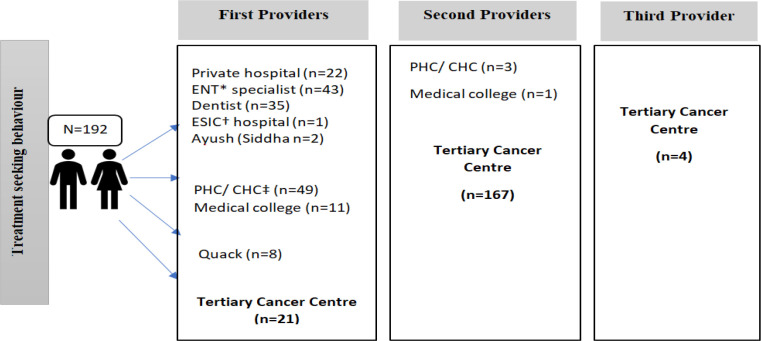
Pathway of the Care among Head and Neck Cancer Patients at Tertiary Cancer Centre (N=192); *Ear Nose Throat Specialist, † Employees’ State Insurance Corporation, ‡Primary Health Centre/ Community Health Centre

## Results

A total of 192 patients out of the eligible 195 were recruited and the response rate was 98%. Majority of the Head and Neck cancer patients were aged between 45-59 years (90, 46.9 %), male (128, 66.7 %), belonged to rural areas (133, 69.3 %), unemployed (142, 74.0 %), and belonged to lower middle class (77, 40.1 %). The common sites of cancer were oral and oropharynx (146, 76 %) and majority reported with stage IV cancer (124, 64.6 %) as shown in Table 1.

The median days (IQR) of the presentation interval, diagnostic interval, and treatment initiation interval were 36.5 (16 - 65.7), 14 (7 - 31.5), and 65.5 (45 - 104) days respectively as shown in Table 2.

Majority (87%) of the patients visited at least one health care provider before reaching the department. The private sector clinic/ hospitals was preferred by 52 % of the patients for initial consultation (Figure 1). 

Definite diagnosis of cancer was made in our tertiary care facility for almost 90% of cases. The median (IQR) of total direct cost, among those who had ever spent for their treatment services, was INR 2400 (700-7300). This estimated total cost in private facilities was spent over a median (IQR) period of 7 (1-20) days. The median direct cost incurred by head and neck cancer patients in our centre and other government facilities were nil. 

Table 3 shows the expenditure on food and lodgement by patients and their caregivers during their diagnosis and treatment in different facilities. 

The patients and their caregivers had soughted care in the studied health care facility for a median period of 240 days and the total median (IQR) wage loss was INR 18000 (5250-61575) during this period. The major source of expenditures were from family savings (56%), the sale of assets (22%) and borrowed (22%). 

The mean Quality of Life (QOL) score was lowest for functional well-being, which was categorized as severely impaired. Other domains of QOL (i.e., Physical, social, emotional, and specific to head and neck) and summary scales (FACT-G and FACT HandN) mean scores were in the moderate range. Better QOL was significantly associated with occupation, when the patient was the head of the family, site and early stage of cancer (Table 4).

Discussion: The present study highlighted the treatment-seeking behavior, treatment cost, and quality of life of the head and neck cancer patients. The majority of the patients were male, age more than 45 years and reported with oral and oropharyngeal cancer in the advanced stage (III and IV) which was similar to various other studies in India (World Health Organization, 2014a; Deka et al., 2015; Mohanti et al., 2007). The preferred first contact for seeking care was the private sector (54%) followed by the government sector (30%). This finding is in contrast to an earlier study conducted in five hospitals across India, which had reported that the cancer patient’s interest and faith were more inclined towards the government sector (47%) than private (45%) (Joshi et al., 2014). Around 11 % of the patient reported to our centre directly; another study conducted in cancer hospital also reported that lesser proportion of study patients (7%) initially report to cancer hospitals (Kumar et al., 2012). The first point of contact for the one-fourth of the patients was the primary/ community health center. The definite diagnosis of cancer was done in our tertiary care centre for 90% of the cases. This may be due to the fact that ours is a regional cancer centre which is well equipped for making the diagnosis. People also visit the center foreconomical reasons, as diagnosis and treatment is almost free at this center. 

The median presentation interval, diagnostic interval, and treatment initiation interval in our study were found 36.5, 15, and 66 days. A study conducted in another comparable centre as ours also reported similar findings; except that the diagnostic interval was two-time more there as compared to our study (Kumar et al., 2012). 

All direct costs related to consultation, diagnosis, and treatment are free of cost in our centre. The patients from the nearby state of Tamil Nadu avail service through the Chief Minister’s Comprehensive Health Insurance Scheme (CMCHIS), under which individuals belonging to annual income less than INR 72000 (~1006 $) can avail free treatment services. The majority of the study patients (82%) were from Tamil Nadu state and from low socio-economic status; insured by CMCHIS (“Chief Minister’s Comprehensive Health Insurance Scheme,” n.d.). 

The average direct cost ever spent by patients in public (7 patients) and private facilities (89 patients) was INR 1000 (400-5,000) and 1,600 (500-6,533) for average of 14 and 7 days treatment respectively. The average direct cost ever paid to quacks was INR 5000 (725-7,500) in average 30 days of seeking care. The out of the pocket expenses during treatment was mostly because of indirect cost. The study on economic burden of cancer conducted in similar setting in Delhi also observed that approximate 60% of the patient expenditure was on transportation, food and lodging during the treatment (Mukhopadhyay et al., 2011). 

A study among 508 cancer patients (all types of cancer) conducted in tertiary care centers of five major cities (Aizawl, Bikaner, Kolkata, Thiruvananthapuram, and Mumbai) of India in 2011 showed that the mean cost of investigation, treatment and indirect expenses over a period of one-year was INR 16739, INR 41311and INR 27248 respectively (Nair et al., 2013). In our study the indirect cost incurred by patients was comparatively less (one-third). The difference may be due to concessional transport scheme available for patients who came from Tamil Nadu and approximate 72 % of the participants were in their first year of treatment. 

A study among 100 oral cancer patients in a private tertiary hospital, stated that the direct costs varied according to stages of oral cancer, and the total direct cost was INR 146092 (72401- 228919) which is much higher as compared to our study (Goyal et al. 2014). Treatment cost estimated in our study may not be representative to head and neck cancer patients seeking care in a private setting.

Considering Quality of Life (QOL) of the patients, there was severe impairment in functional wellbeing whereas there was moderate impairment in other dimensions. A study in All India Institute of Medical Sciences, Delhi stated that functional scores decline during the treatment and for those having symptoms related to disease (like pain, fatigue, nausea etc.) which increase during the course of the treatment (Bansal et al., 2004). The patients were were employed, were head of their family and were in early stage of nasal, nasopharyngeal, parotid and thyrod cancer had significantly better QOL than other cancer patients. The study from Karachi, Pakistan which used same scale (FACT-G and HandN scale) also found that there was significant association between occupation, stage and site of cancer with QOL (Bilal et al., 2015). We could not find significant association between QOL and various demographic variables and socioeconomic status. In contrast to our study, a study conducted at Regional Cancer Centre, Trivandrum using the FACT-G scale, reported that patients with higher socioeconomic status had better QOL (Thomas et al., 2004). 

*Limitation*

In our study, the patients who were not able to speak due to their illness, their QOL assessment was done based on the responses of their caregiver; thus, there is a possibility of information bias. Patients were asked the about their symptom recognition and pathway of care, there are chances of having recall bias. Since the study was conducted in the hospital setting it is expected that the characteristic of cancer patients seeking care from hospital may be different from that of cancer patients in the community due to berksonian bias. Treatment cost estimated in our study may not apply to all head and neck cancer patients, especially those who are seeking care in a private setting.

*Strength*

The present study tried to identify the important aspects of the treatment of head and neck cancer patients. Validated Tamil version of the tool was used for collecting information related to the quality of life of patients.

In conclusion, preferred healthcare provider was private sector as reflected in the pathways of care and majority of patients visited at least one provider before reaching the tertiary care facility. The average treatment initiation interval was more than two months. The expenditure was mostly on indirect cost and initially patients/ their caregivers spend from their own savings, but at a later stage, they start selling their assets and ultimately landed-up borrowing money for their treatment. Their overall quality of life was moderately impaired. 

Screening and referral mechanism at primary/ community health centers can reduce the presenation time interval as has been already initiated by the National Programme for prevention and Control of Cancer, Diabetes, Cardiovascular Diseases and stroke. Further research is needed to understand the physical, social, familial, and functional quality of life to different disease parameters.

## Author Contribution Statement

None.
